# An incremental dual-task paradigm to investigate pain attenuation by task difficulty, affective content and threat value

**DOI:** 10.1371/journal.pone.0207023

**Published:** 2018-11-09

**Authors:** Quoc C. Vuong, Angela Owen, Kehinde Akin-Akinyosoye, Vera Araujo-Soares

**Affiliations:** 1 Institute of Neuroscience, Newcastle University, Newcastle upon Tyne, United Kingdom; 2 Arthritis Research UK Pain Centre, University of Nottingham, Nottingham, United Kingdom; University of Rome, ITALY

## Abstract

There is accumulating evidence that task demands and psychological states can affect perceived pain intensity. Different accounts have been proposed to explain this attenuation based either on how limited attentional resources are allocated to the pain stimulus or on how the threat value of the pain stimulus biases attention. However, the evidence for both proposals remains mixed. Here we introduce an incremental dual-task paradigm in which participants were asked to detect pain on their fingertip without any additional tasks during baseline phases or while concurrently detecting visual targets during task phases. The force applied to participants’ fingertip in all phases increased incrementally until they detected moderate pain. In Experiment 1, we used coloured shapes and in Experiment 2 we used affective images as visual targets. We also manipulated the threat value of the pain stimulus in Experiment 2. For both experiments, we found that a concurrent task attenuated perceived pain intensity: mean force was significantly greater for the same moderate pain during task compared to baseline phases. Furthermore although task difficulty and affective content did not affect pain perception, the threat value of the pain stimulus moderated the magnitude of pain attenuation.

## Introduction

People’s perception of pain intensity from noxious stimulation (e.g., from heat, cold or mechanical force) are often affected when they are engaged in a cognitively or emotionally demanding task (e.g., [1–12]; for a review see [13]). There are two prominent accounts to explain the modulation of perceived pain intensity when people are performing a task. Both are predicated on how attention is allocated to the noxious stimulation or to the task at hand, which subsequently affects how people process the stimulation and judge the perceive intensity of the resulting pain signals. In the first account, researchers propose that task processing and pain processing compete for limited attentional resources; we will refer to such proposals as “attentional-capacity models” of pain attenuation [14–17]. According to such models, task-demand factors (e.g., difficulty or working-memory load) are the critical factors that influence the perceived pain intensity of noxious stimulation; such factors recruit limited attentional resources leaving fewer resources to process pain signals. Second and more recently, researchers propose that psychological factors—such as people’s emotional and arousal state, motivation, and how threatening they perceive the noxious stimulation—bias their attention towards or away from pain signals even if attentional resources are available for both task and pain processing; we will refer to such proposals as “attentional-bias models” of pain attenuation [18–23].

Although both attentional-capacity and attentional-bias models provide an appealing explanation of how various factors attenuate perceived pain intensity, which model best captures the mixed experimental findings continues to be debated [19, 24–26]. That said, these models may not necessarily be mutually exclusive. Thus the primary scientific aim of the current study was to better understand the relationship between these two models to clarify how task-demand and psychological factors affect pain perception. Towards that end, we present a novel incremental dual-task paradigm [27] to assess how task difficulty (attentional-capacity models), and affective content and threat value of noxious stimulation (attentional-bias models) affect perceived pain intensity. This paradigm emulates real-world situations and allows task-demand and psychological factors to be systematically manipulated in a single paradigm.

There is a large body of evidence to show that engaging in a task attenuates the perceived pain intensity of different noxious stimulations; for example, as measured by subjective numeric ratings or visual analogue scales (for a review see [13]). However, several researchers note that such evidence for attentional-capacity models of pain attenuation remain inconclusive [19, 24], particularly as performing a task does not always reduce perceived pain intensity [28–32]. The discrepancies between previous studies has been attributed to potential methodological issues [4, 7, 33–36]. For example, participants often need to evaluate experimentally-induced pain which has a fixed intensity while they perform a task, so that they can subsequently assign a numeric value to rate their pain experience. This pain-evaluation process may change over time as participants adapt to the fixed experimental pain level leading to different findings depending on the delay duration. Moreover, the delay between experiencing the noxious stimulation and rating the resulting pain may lead to retrospective errors or biases [34–36].

Attentional-bias models of pain attenuation were proposed partly to address in two key ways some of these mixed findings reported in the literature. First, such models incorporate the affective content of the stimuli related to the task rather focusing solely on task-demand factors. The affective content can affect observers’ emotional and arousal states, and subsequently bias their attention to such stimuli rather than recruit attentional resources *per se*. In line with this, it has been shown that images with negative valence affects visual-search performance (e.g., [37]). Similarly, there is evidence that images with different affective content (e.g., positive versus negative valence images) affect perceived pain intensity of different types of noxious stimulation (e.g., [2–3, 38–39]; for a review see [13]). However, the effect of affective content on perceived pain intensity is mixed. For example, Peláez et al. [38] showed that subliminally presented negative emotional images decreased perceived pain intensity, whereas Kenntner-Mabiala et al. [39] showed that viewing (supraliminal) negative emotional images increased perceived pain intensity. Second, attentional-bias models also incorporate the threat value of the noxious stimulation which—like affective content—can bias observers’ attention to pain signals rather than recruit attentional resources *per se*. Researchers particularly emphasise that if people perceive a noxious stimulation to be threatening, they are more likely to attend to pain signals and may find it more difficult to disengage their attention from them. In these studies, the perceived threat value is often manipulated by instructions emphasising (or not) the potential physical harm from that noxious stimulation. For example, McGowan et al. [40] found that increasing the threat value of cold stimulation increased subjective pain ratings. By comparison although Van Damme et al. [41] found that increasing the threat value of cold stimulation increased various psychological measurements (e.g., anxiety) and decreased task performance, it did not affect pain ratings (see also [25]). Other studies found that threat value affected how people disengaged attention from pain signals [42–43] or learned pain associations [44]. A recent study found that threat value did not differentially affect those with or without chronic pain [45]. Thus across the studies reviewed the effects of factors such as task difficulty and threat value on pain perception and whether these factors interact remains unclear.

**Fig 1:**
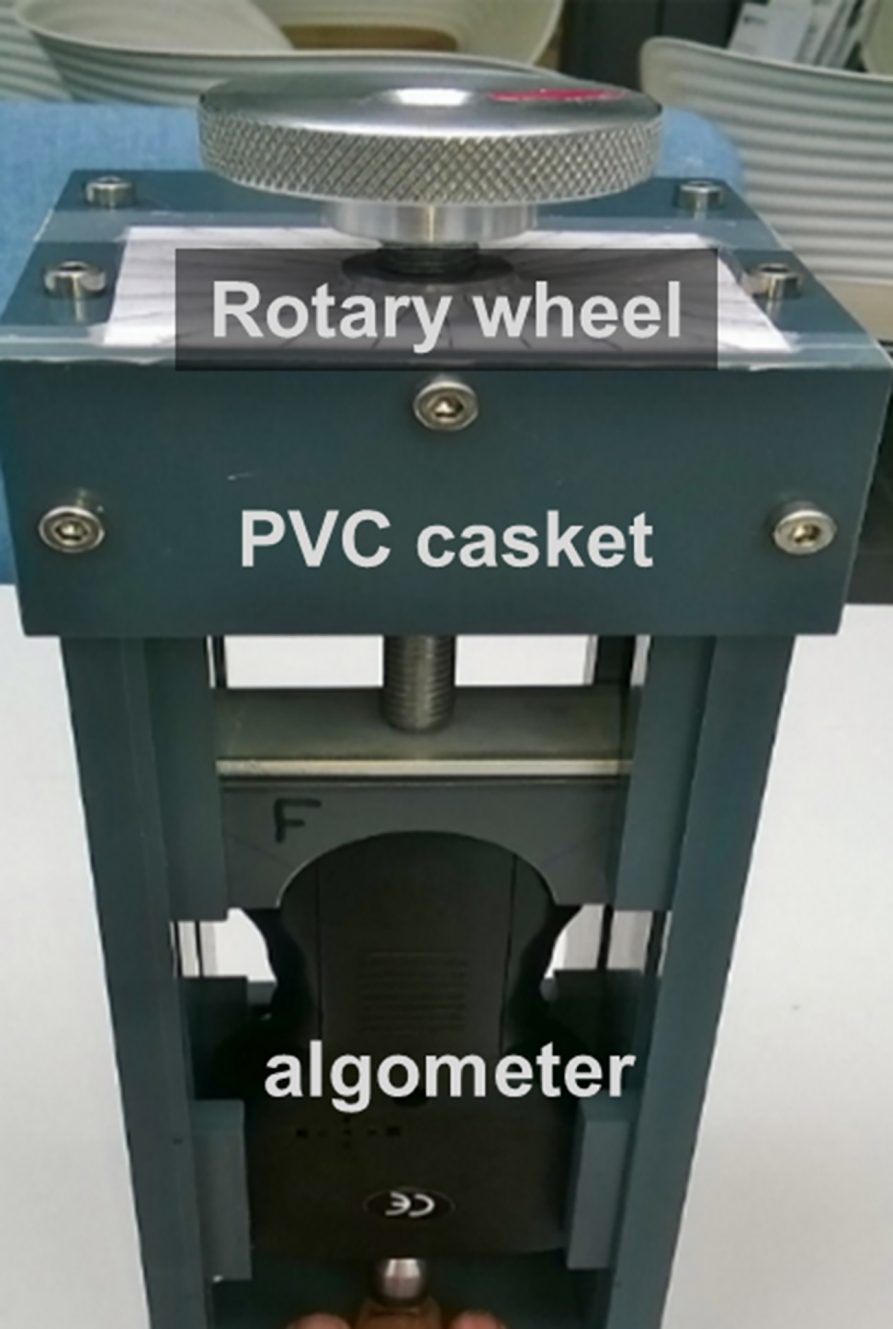
The force pain-induction device. Illustration of the pain-induction device from the participant’s point of view. A digital algometer is placed in a custom-built PVC casket which has a rotary wheel on top. A rotation scale is printed below the metal wheel to allow controlled amount of rotation.

We sought to address these discrepancies and possible methodological limitations by using the same pain-induction method and the same experimental paradigm inspired from the attention literature in two experiments [27, 46–47]. In our incremental dual-task paradigm, we measured and compared the force (measured in Newton) applied to the fingertip during baseline phases in which participants were instructed to *only* detect moderate pain at their fingertip (i.e., single-task condition) and during a task phase in which they were instructed to *concurrently* detect moderate pain *and* visual targets (i.e., dual-task condition) without compromising performance on the visual task. There was a baseline phase before and after the task phase. In all phases, we apply force to participants’ fingertip in small incremental steps (from no force, i.e., no pain) using a custom built device (Fig 1; [48]). On task phases, the pain and visual-target detection tasks can be performed concurrently either by dividing attention between the pain and visual stimuli or by switching attention between each stimulus type. We investigated whether and how factors like task difficulty, affective content and threat value of the noxious stimulation influenced these processes but note that our paradigm allows for any variety of manipulations. This paradigm also approximates real-world situations in which the pain intensity may develop over short time periods or when moderate pain may occur unexpectedly during the course of people’s daily activities. There is evidence that acute and chronic pain can impair performance on similar divided-attention and task-switching paradigms [11, 31, 49–50]. However, no studies have investigated how such paradigms affect pain perception nor have they treated pain detection as a “task” *per se*. Importantly for our purposes, dual-task paradigms assume that if performance on one or the other task is affected by performing both tasks then the two tasks share attentional resources [27].

In Experiment 1, we manipulated task difficulty while participants performed a visual working-memory task during the task phase [10, 51]. In Experiment 2, we manipulated both affective content and threat value while participants performed a visual-search task during the task phase. Both of these psychological factors can bias attention toward or away from the noxious stimulation. If task and pain processing share limited resources (i.e., attentional-capacity models), then we predict that participants will tolerate more force for the same perceived moderate pain intensity when they are concurrently engaged in a visual task, and that task difficulty will moderate pain attenuation. If threat value biases attention to the pain stimulus (i.e., attentional-bias models), then we predict that participants in the high-threat compared to low-threat group would have a smaller attenuation of perceived pain intensity by the task. The threat value of the force stimulus may further interact with the affective content of the images.

## Materials and methods

### Force pain induction

Fig 1 shows the custom-built pain-induction device used to apply force stimulation to the fingertip [48]. The device consists of a digital algometer (FDX Force 25; model FDX5; Wagner Instruments, Inc.) housed in a PVC casket. It has a metal rotary wheel with markings every 15°. There is good reliability of pressure pain thresholds measured with algometers in healthy young pain-free volunteers and clinical populations at anatomical sites (e.g., [52–54]).

During pain induction, participants placed their left hand palm-side down in the casket with the middle fingertip below the algometer’s circular rubber tip (1 cm diameter). The tip was lowered until it rested on the fingertip and then the algometer was tared; this was designated as the rest position (0 N). The experimenter incrementally applied force to the fingertip at a constant rate by rotating the wheel 15° every 2.0 s, using a digital metronome to maintain this rate (tempo = 30 beats/min). Participants were not informed of this constant rotation rate.

### Experiment 1

#### Participants

**Fig 2:**
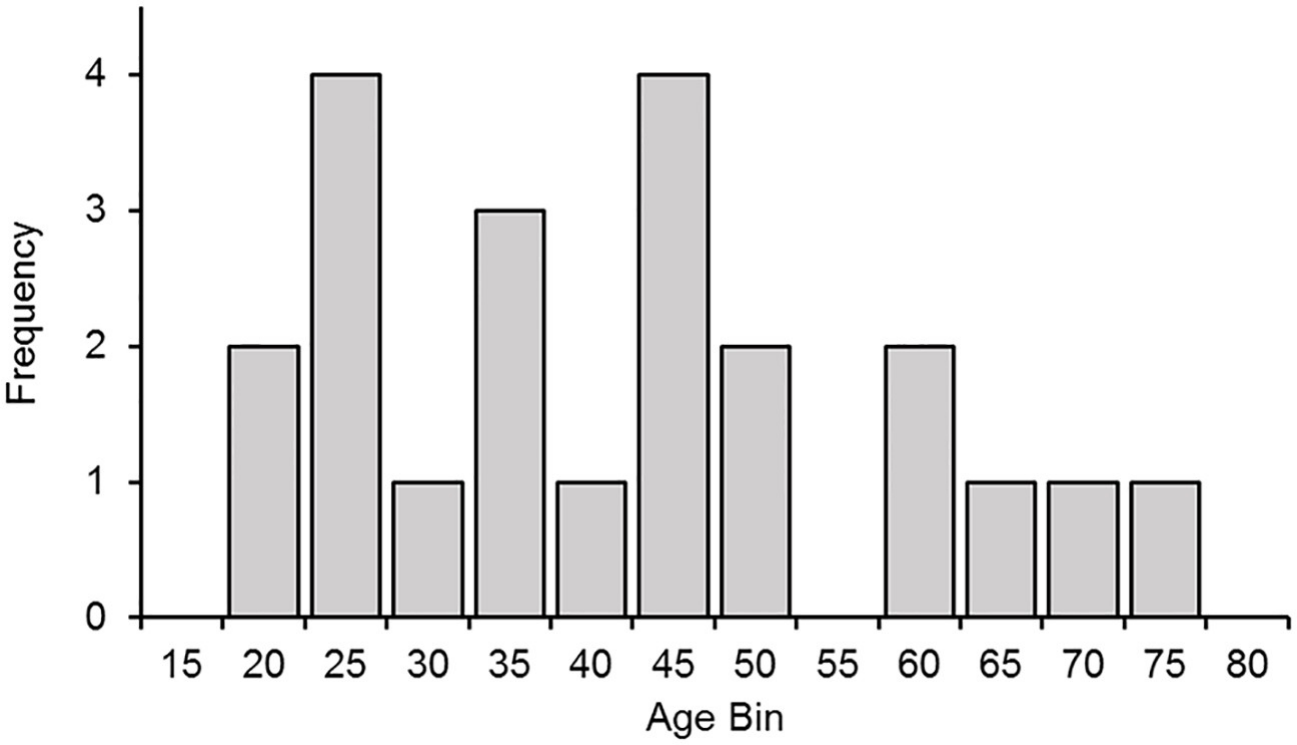
Age distribution in Experiment 1. Frequency of participants in each age bin (Bin 25 = 21–25; Bin 30 = 26–30; Bin 35 = 31–35; etc.).

Twenty-two naïve volunteers participated in Experiment 1 (13 females; age: 20–74 years; *M* = 39.6 years, *SD* = 16.4 years). Fig 2 shows the histogram of age distribution. All participants in this and the subsequent experiment were recruited from a volunteer database or an undergraduate research participation scheme, and were naïve to the purpose of the present study (i.e., that we were testing how task difficulty, affective content and threat value affected perceived pain intensity). They self-reported that they were in good physical health; had normal or corrected to normal vision; had no known colour blindness; and had no known history of psychological, neurologic, or psychiatric disorders.

The ethics for the experimental protocols used in this study was approved by the Faculty of Medical Sciences Ethics Committee at Newcastle University. The research was conducted in accordance with institutional guidelines and regulations. All participants provided written informed consent (including those for the online questionnaire). Participants in Experiments 1 and 2 were paid for their participation or received course credit.

#### Stimuli and apparatus

The visual stimuli used in Experiment 1 consisted of 16 coloured shapes created from the factorial combination of four shapes (circle, diamond, triangle and hexagon) and four colours (red, green, blue and yellow). Participants were tested in a dimly lit room. They sat approximately 60 cm away from a flat-panel monitor (1280 x 1024 pixel resolution, 60 Hz refresh rate). The experiment was controlled by a Windows PC using the Psychtoolbox [55–57] extension for Matlab (64-bit; 2012b version; Mathworks, Inc).

The experimenter sat opposite and to the left of the participant throughout the experiment so that the experimenter was not directly in front of participants. The pain-induction device was placed between them at a comfortable distance for the participant. The experimenter manually recorded the force measurement from the algometer’s digital display. Both the measurements and digital display was visible only to the experimenter. The experimenter also wore headphones connected to the metronome to ensure that participants could not hear the metronome’s beat.

#### Design and procedure

Participants were tested in three sequential phases: a pre-task, task and post-task phase (in that order). Both the pre- and post-task phases are baseline phases (i.e., single-task condition or no concurrent visual task). The pre-task phase began with participants comfortably seated and the pain-induction device in the rest position. The aim of this phase was to provide participants with the opportunity to experience a range of pain intensity from the force stimulation, and particularly to experience the moderate pain which served as the target pain intensity for the remainder of the experiment. The experimenter first explained to participants that they would report different pain intensity using a numeric rating scale (from 1 to 10, not including 0) as force was incrementally applied to the fingertip. They were instructed to verbally indicate Level 1 when they first experienced some pain (pain threshold); to indicate subsequent numeric ratings up to Level 9 each time they experienced an increased level of perceived pain intensity relative to the previous rating; and to indicate Level 10 (pain tolerance) when they could not tolerate any more force. After experiencing all intensity levels, the experimenter informed participants that the moderate pain intensity (Level 5) would be the pain intensity to monitor for during the task and post-task phases. The procedure for the post-task phase was the same as the pre-task phase except that participants were only asked to report when they experienced the moderate pain intensity (Level 5). One block was conducted in the pre-task phase (from Levels 1 to 10) and two block were conducted in the post-task phase (only at Level 5). Thus the post-task phase measured force in a manner more similar to the task phase (i.e., a single measurement at moderate pain intensity).

During the task phase, participants were instructed to monitor the force applied to their left fingertip for a moderate pain intensity (i.e., Level 5) *and* to perform a working-memory task as quickly and as accurately as possible. There were two difficulty levels for the working-memory task, easy (1-back) or hard (3-back), which were run in alternating blocks. There were four repetitions of each difficulty level, resulting in eight blocks for the task phase. Whether testing started with the easy or hard level was counterbalanced across participants. All participants did 10–15 practice easy trials without any pain induction (but with their left hand in the pain-induction device) to become familiar with the working-memory task.

Participants pressed the space bar which started each block and signalled to the experimenter to begin rotating the wheel from the rest position. They were instructed to press the space bar again with their right hand as soon as they experienced a moderate pain intensity at their fingertip; this terminated the block. Concurrently to the force monitoring, they performed as many working-memory trials as possible. For each trial, participants were shown a sequence of coloured shapes. Their task was to determine if the current target stimulus matched both the shape and colour of the stimulus in the preceding *n* items back (i.e., 1-back or 3-back) in the sequence. Each target stimulus was presented at the centre of a grey screen, and remained on the screen until participants responded by pressing the “match” or “no-match” key with their right hand as quickly and as accurately as possible. Each stimulus in the sequence was randomly selected with replacement from the 16 possible stimuli with the constraint that both “match” or “no-match” trial types were equally likely for each stimulus presented. Response times were measured from the onset of a stimulus. If participants responded incorrectly, they heard a 2500 Hz tone for 500 ms through headphones. If they responded correctly there was no tone. The response keys were counterbalanced across participants. Following the participants’ response, there was a 500 ms grey screen before the next target was presented. There was an enforced 2 minute break after each block to reduce pain sensitization.

### Experiment 2

#### Participants

Thirty-one naïve volunteers participated in Experiment 2 (23 females; age: 18–23 years, *M* = 19.8 years, *SD* = 1.4 years). Sixteen participants were randomly assigned to the low-threat group and 15 were randomly assigned to the high-threat group.

#### Stimulus and apparatus

The images used in Experiment 2 consisted of 48 images equally divided into positive, neutral, negative and discomfort image sets. The positive, neutral and negative images were selected from the International Affective Picture System [58] based on their arousal and valence ratings. The discomfort images were downloaded from the internet and showed a single person in some form of pain or distress (e.g., an adult male sitting on his bed and clasping his stomach from stomach pain; see S1 Text). All images were centred within a 256 pixels x 256 pixels black square. Otherwise, the same apparatus and setup as in Experiment 1 were used in this experiment.

#### Design and procedure

In Experiment 2, participants were randomly assigned to two different groups. Participants in the high-threat group were informed that “exposure to pressure can cause some bruising, this may be associated with numbness and pain in the tingling finger”. Those in the low-threat (neutral) group were informed that “exposure to pressure is harmless, but can be associated with some pain and discomfort; this is absolutely normal and has no further consequences.” This threat manipulation procedure was adapted from Van Damme et al. [41].

Participants in both groups were tested in the same pre-task, task and post-task phases. The pre-task and post-task phases were the same as in Experiment 1, with the exception that two blocks were conducted for both the pre-task (from Levels 1 to 10) and post-task (only Level 5) phases. During the task phase, participants were instructed to monitor their left fingertip for a moderate pain intensity *and* to perform the visual-search task as quickly and as accurately as possible. The four image sets (positive, neutral, negative and discomfort) were run in separate blocks and each image-type block was repeated three times. Each participant therefore completed a total of 12 blocks during the task phase. For each participant, image sets were run in a different pseudo-random order. All participants completed 10–15 practice trials using the neutral image set without any pain induction (but with their left hand positioned within the pain-induction device) to familiarize themselves with the task.

The procedure for the task phase was the same as in Experiment 1, with the exception that participants performed a visual search task rather than a working-memory task. Each search trial began with a white fixation cross presented in the centre of a grey screen for 500 ms, followed by a target image presented at the centre of the screen for 1000 ms, followed by a blank screen for 500 ms, and lastly followed by a 3 x 3 grid of images distributed evenly across the screen. The grid remained on the screen until participants responded. For each trial, the target and grid images were randomly selected from the 12 possible images for that set. The participants’ task was to determine if the target image was present or absent in the grid by pressing the “present” or “absent” key as quickly and as accurately as possible. The response mapping was counterbalanced across participants. Present and absent trial types were equally likely. On present trials, the location of the target image was randomly selected from the nine possible locations in the grid. Response times were measured from the onset of the grid. As in Experiment 1 if participants responded incorrectly, they heard a 2500 Hz tone for 500 ms through headphones. Following the participants’ response, there was a blank screen for 500 ms before the next trial began. As in the first experiment, participants were instructed to press the space bar with their right hand as soon as they experienced a moderate pain intensity on their left fingertip; this terminated the block. There was an enforced 2 minute break after each block to prevent pain sensitization.

Following previous work (e.g., [41]), we administered different questionnaires at the end of Experiment 2 to measure additional psychological factors that may correlate with pain measurements. These questionnaires include the Pain Catastrophizing Scale (PCS; [59]), the Toronto Empathy Scale (TEQ; [60]), the Fear of Pain Questionnaire (FPQ; [61]) and the Depression, Anxiety and Stress Scale (DASS; [62]) (in that order).

## Results

### Experiment 1: Working memory

#### Behavioural data

**Fig 3:**
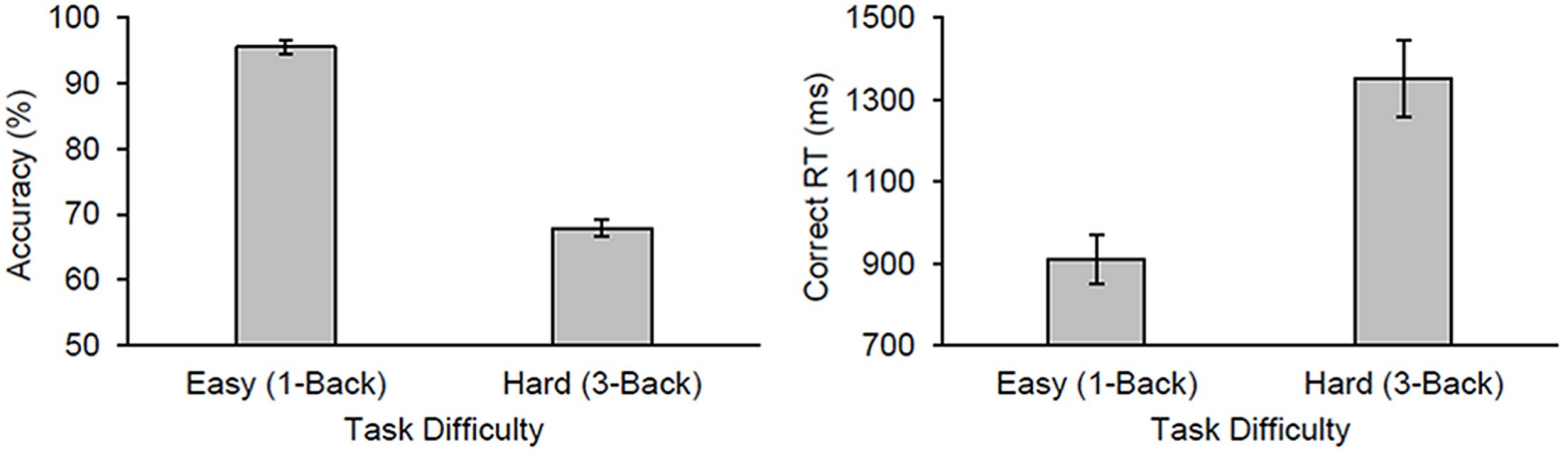
Behavioural results from Experiment 1. The mean accuracy (percentage correct) and correct response times (RTs) as a function of difficulty level in Experiment 1. The error bars reflect the standard error of the mean in this and subsequent figures.

Participants completed a mean of 31.9 trials/block (*SE* = 3.5 trials/block) before terminating a block (duration per block: *M* = 48.6 sec, *SE* = 4.0 sec). Fig 3 shows the mean accuracy (percentage correct) and response times (RTs) from correct trials for each difficulty level. Participants responded more accurately (*t*(21) = 17.70, *p* < .001) and more quickly (*t*(21) = 6.59, *p* < .001) on the easy compared to the hard level of the working-memory task. Thus task difficulty reduced performance.

#### Force measurement at moderate pain

**Fig 4:**
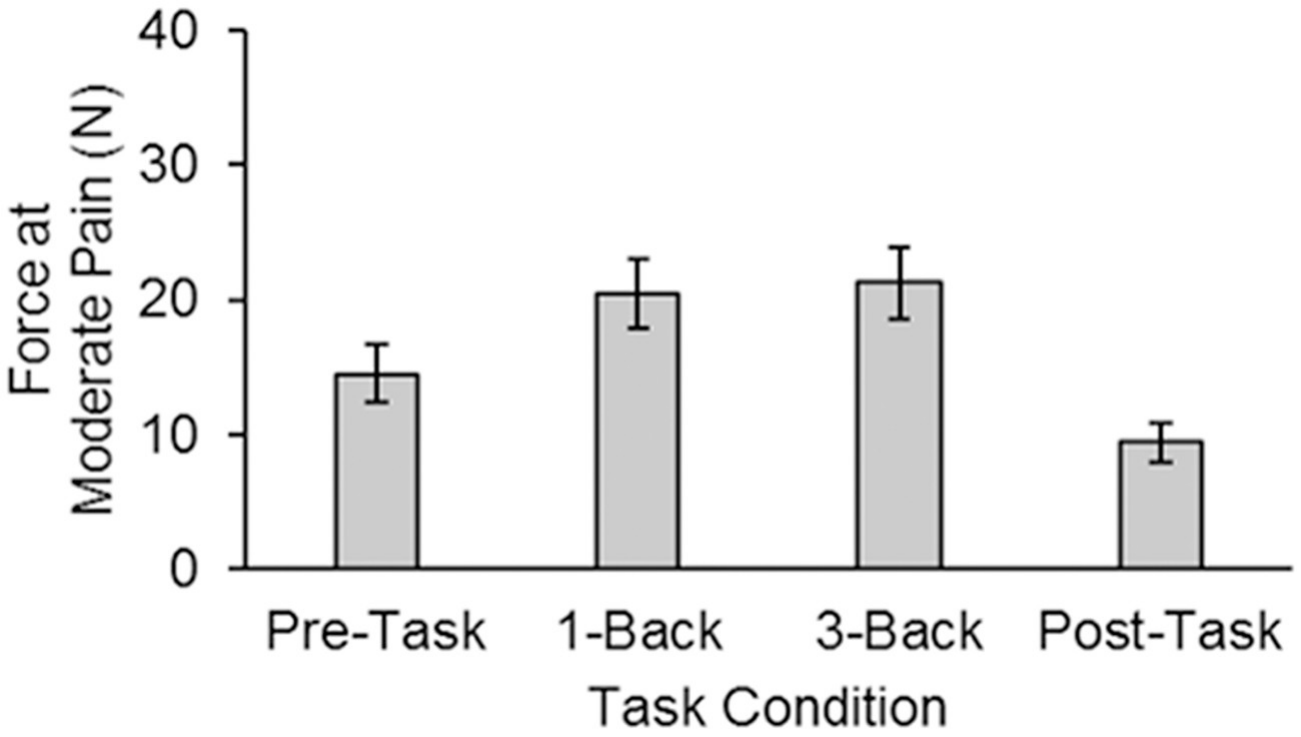
Force results from Experiment 1. The mean force at moderate pain (Level 5) as a function of task condition in Experiment 1.

Fig 4 shows the mean force at moderate pain (Level 5 out of 10) for the differerent task conditions in Experiment 1. We first compared the two difficulty levels (run on alternating blocks during the task phase), which showed that there was no significant difference in mean force between these conditions, (*t*(21) = 1.31, *p* = .27). We therefore averaged across difficulty level. Because the three phases were run in a fixed order, we could not use an analysis of variance (ANOVA) which assumed that the phase factor was randomly ordered for each participant. Rather we conducted pairwise *t*-tests. These pairwise comparisons showed that participants had a greater mean force (i.e., tolerated more force for the same perceived moderate pain intensity) during the task phase (*M* = 20.9 N, *SE* = 2.6 N) compared to the pre-task (*t*(21) = 6.13, *p* < .001) and post-task (*t*(21) = 9.48, *p* < .001) phases. Interestingly, they also tolerated more force in the pre-task compared to the post-task phase, *t*(21) = 5.17, *p* < .001, which may reflect some pain sensitization despite an enforced recovery period. Overall the task and baseline force measurements differed by 8.9 N on average, which reflects a 28.3% attenuation in perceived pain intensity when participants were engaged in a visual working-memory task. This attenuation was computed as:

$$\%attenuation=\frac{N_{task}-N_{baseline}}{N_{task}+N_{baseline}}\times 100$$

where *N*_*task*_ is the mean force at moderate pain for the task phase and *N*_*baseline*_ is the mean force at moderate pain for the baseline phase (averaged across the pre- and post-task phases).

### Experiment 2: Affective content and threat value

#### Behavioural data

**Fig 5:**
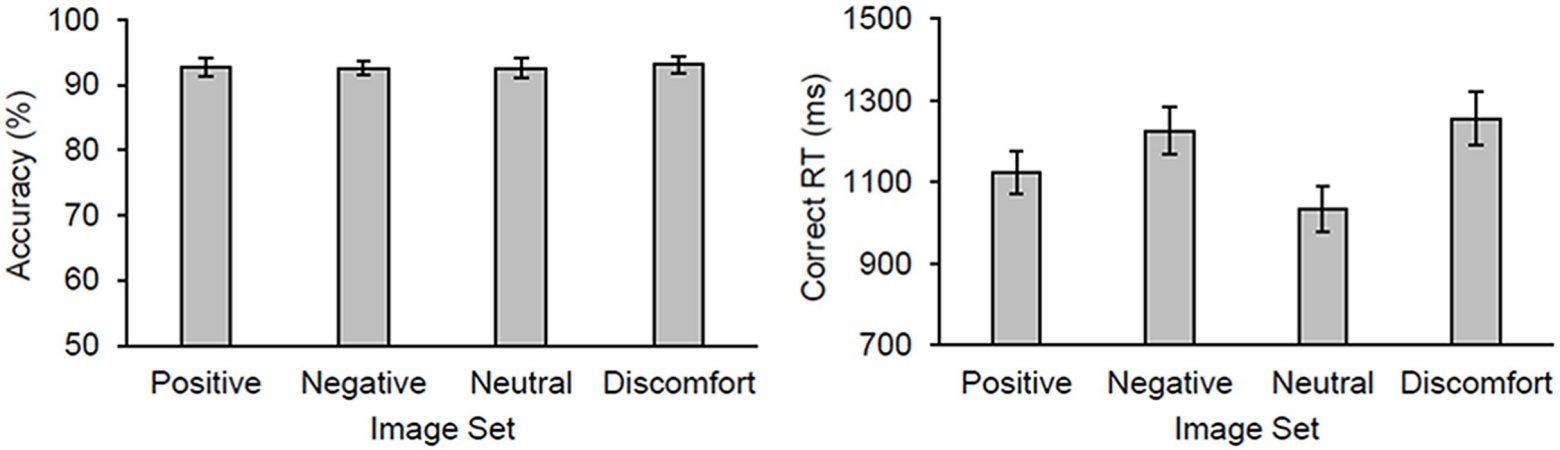
Behavioural results from Experiment 2. The mean accuracy (percentage correct) and correct response times (RTs) as a function of image set in Experiment 2.

Participants completed a mean of 11.0 trials/block (*SE* = .7 trials/block) before terminating a block (duration per block: *M* = 41.1 sec, *SE* = 2.7 sec). Fig 5 shows the mean accuracy and correct RTs as a function of image set. We submitted the behavioural data to a 2 x 4 mixed ANOVA with threat group as a between-subjects factor and image set as a within-subjects factor. Overall participants responded very accurately (>91% in all conditions). There was no main effect of threat group nor image set on accuracy, *F*s < 1.0, and there was a marginal interaction between these two factors, F(3,87) = 2.27, *p* = .09, $\eta_{p}^{2}=.07$. By comparison, for correct RTs there was a significant main effect of image set, *F*(3,87) = 25.11, *p* < .001, $\eta_{p}^{2}=.46$, but no main effect of threat group, *F*(1,29) = 1.94, *p* = .17, $\eta_{p}^{2}=.06$, and a marginal interaction between threat group and image set, *F*(3,87) = 2.40, *p* = .07, $\eta_{p}^{2}=.08$. Post-hoc comparisons collapsing across group showed that participants responded quickest with neutral images followed by positive images, and they responded slowest with both negative and discomfort images (*p*s < .002, uncorrected). There was no significant difference between the negative and discomfort image sets. Thus the affective content of the images slowed responses, with negative and discomfort images impairing response time the most.

#### Force measurement at moderate pain

**Fig 6:**
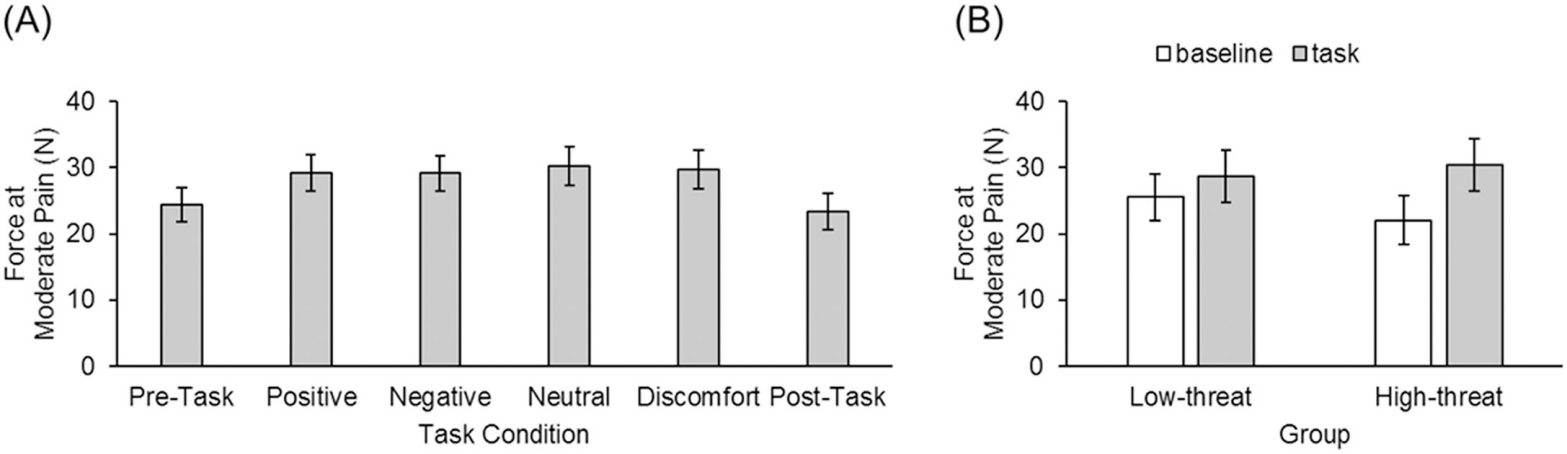
Force results from Experiment 2. The mean force at moderate pain (Level 5) in Experiment 2. (A) The mean force as a function of task condition pooled across the low-threat and high-threat group. (B) The mean force as a function of group and phases (baseline and task).

Fig 6 shows the mean force at moderate pain as a function of task condition in Experiment 2. We conducted several preliminary analayses to help interpret the results. First, we submitted the force data from the task phase to a 2 x 4 mixed ANOVA with threat group as a between-subjects factor and image set (run in alternating blocks during the task phase) as a within-subjects factor. This analysis showed no significant main effects of group and image set, and no significant interaction between these two factors, all *F*s < 1.58, *p*s > .20. We further compared the pre- and post-task phases across all participants and found no difference between these phases, *t*(30) = .60, *p* = .55, suggesting that no pain sensitization occurred in this experiment. As in Experiment 1, we conducted pairwise *t*-tests to further investigate differences across conditions, pooling the data across image sets (during the task phase) and across pre- and post-task phases. We found no significant difference in force measurements between the high-threat and low-threat groups, *t*(29) = .17, *p* = .87, and a significant difference in force measurement between the task and baseline phases, *t*(30) = 5.94, *p* < .001. Furthermore for participants in both groups, there was a significant difference between the task and baseline phase (high-threat: *t*(14) = 5.65, *p* < .001; low-threat: *t*(15) = 3.64, *p* = .002). Importantly the *difference* between the task and baseline phase (i.e., pain attenuation by task demands) was larger for participants in the high-threat (*M* difference = 8.5 N, *SE* = 1.5 N) compared to the low-threat (*M* difference = 3.2 N, *SE* = .9 N) group, *t*(29) = 3.08, *p* = .005. The pain attenuation was 9.6% for the high-threat group and 16.0% for the low-threat group; however, this difference was not significant, *t*(29) = 1.68, *p* = .10. Across both groups the task and baseline force measurements differed by 5.7 N on average, which reflects a 12.7% reduction in perceived pain intensity when participants were engaged in a visual-search task.

#### Psychological measurements

Table 1 presents the mean for the low and high threat groups (pooling across subscales). There were no significant differences between the two groups on any of the questionnaire scales (or their subscales). Only the fear-of-medical-pain subscale of the FPQ [61] significantly correlated with force measurements in the task, *r*(29) = -.43, *p* = .02 and baseline phase, *r*(29) = -.47, *p* = .01. The anxiety subscale of the DASS [62] was marginally correlated with force measurements on both of these phases (task: *r*(29) = -.30, *p* = .10; baseline: *r*(29) = -.31, *p* = .09).

**Table 1:** Results of the psychological measurements for the low-threat and high-threat groups in Experiment 2.

	PCS	FPQ	DASS	TEQ
Low	19.2 (1.5)	26.4 (1.7)	23.6 (5.0)	47.4 (1.4)
High	21.6 (2.6)	23.4 (1.0)	30.3 (6.1)	46.4 (1.7)

Mean (SEM) of the total scores for the different scales as a function of threat group. PCS = Pain Catastrophizing Scale; FPQ = Fear of Pain Questionnaire; DASS = Depression, Anxiety and Stress Scale; TEQ = Toronto Empathy Questionnaire.

## Discussion

Pain serves as an important warning function to alert people of potential bodily harm from noxious stimulation in the environment (e.g., heat, cold or force). Although pain often grabs their attention for this function, there are real-life anecdotes and accumulating empirical evidence that competing tasks and heightened emotional and affective states can often (but not always) attenuate the perceived intensity of the pain from the noxious stimulation [13]. In line with these observations, we found that participants tolerated more force applied to their fingertip for the same perceived moderate pain intensity when they concurrently monitored for a pain stimulus and for visual targets (i.e., dual-task condition) compared to when they only monitored for a pain stimulus (i.e., single-task condition). Furthermore, we found that the magnitude of pain attenuation was moderated by the perceived threat value of the force stimulation [40–41]. Quite surprisingly, this reduction was nearly three times larger when we emphasised the potential physical harm of the pain-induction device (high-threat group) then when we did not (low-threat group). This is unexpected because attentional-bias models would make the opposite prediction that pain attenuation should be larger in the low-threat than high-threat group. Across both groups, we found a correlation only between force measurements and the fear-of-medical-pain subscale of the FPQ [61]. By comparison some studies (e.g., [40]) found that subjective pain experiences correlated with anxiety [62].

Our study was partly motivated by the fact that the experimental evidence is mixed for both attentional-capacity [14–17] and attentional-bias models [19–23, 38–39]. By manipulating task difficulty, affective content and threat value within a single paradigm, our findings suggest that attentional-capacity and attentional-bias models are likely to be complementary. For instance, there may be attentional switching between the pain stimulus and the visual stimulus to deal with limited resources but it may be more difficult to disengage from threatening compared to nonthreatening pain signals [25, 63]. At the same time our findings highlight important limitations of both types of models. For example, attentional-capacity models predict that pain attenuation should vary with task difficulty. We did not find this dependency. Attentional-bias models predict that performing a task should be less effective at pain attenuation if participants perceive the pain stimulus to be threatening (e.g., can cause physical harm; [41, 64]). On the contrary, we found the opposite.

Despite the large general effect of task engagement on the attenuation of perceived pain intensity and its moderation by threat value, there remains limitations that we need to consider. First, we did not find an effect of task difficulty or affective content on pain perception as found in previous studies (e.g., [3, 7, 10, 12]). However, not all studies found that task demand affected pain perception [28–32]. These manipulations did affect performance on the corresponding task, suggesting that were effective in influencing the allocation of attentional resources. In Experiment 1, both accuracy and response times were impaired in the hard (3-back) relative to the easy (1-back) blocks, suggesting that our task-difficulty manipulation effectively influenced how limited attentional resources were allocated to the task. Likewise in Experiment 2, negative and discomfort images increased response times relative to neutral and positive images, suggesting that our affect manipulation biased attention more to images with negative compared to those with positive valence. Arguably, the effects of these manipulations on behaviour were small; for instance, differences in response times between the different conditions were on the order of 200 to 500 ms across the two experiments. Therefore, we may not have made differences between conditions large enough to recruit additional attentional resources beyond the amount initially recruited by the task *per se*. That said, Veldhuijzen et al. [12] showed that participants experienced less pain from cold stimulation after hard compared to easy blocks (~12% reduction; 1.2-cm difference on a 10-cm visual analogue scale) with differences in response times similar to ours on the order of 200 to 300 ms.

Second, we found that pain attenuation by task was significantly larger for the high-threat compared to the low-threat group. One possibility is that observers in the high-threat group engaged in threat-avoidance behaviour by endogenously attending to the visual-search task [65]. Third and lastly, we did not find any significant group differences for any of the psychological measurements. This result is different than Van Damme et al.’s [41] study, in which they reported that participants in the threat group reported more catastrophic thoughts about cold stimulation and were more anxious than those in the neutral group. There was, however, moderate correlations between force measurements and fear of medical pain. Recall that the cover story to manipulate the threat value of the force device was that the algometer could lead to long-term damage to the fingertip. Alternatively, the threat manipulation may have led to short-term discomfort.

These limitations are balanced by several strengths of our novel incremental dual-task paradigm. The key strength is that we treated pain perception as a task *per se*, which better reflects real-world situations in which people do “detect” noxious stimulation while engaged in their daily activities (e.g., to avoid bodily harm). This allowed us to both exploit dual-task paradigms [27] and move away from subjective pain evaluations and ratings. Second, our paradigm allowed us to manipulate both task demands and psychological factors within a single paradigm. It would be important, therefore, in future studies to more systematically investigate how task demands and threat value interact with our new paradigm For example, we can increase task demands or valence level between conditions (e.g., select images with more extreme valence levels; [58]) as the findings may have implications for behavioural pain-management strategies. It would also be important to separate emotional from affective influences (which was confounded in our study).

## Conclusions

There is clearly a need for more research in this area to unravel whether and how attention is allocated to deal with potentially harmful noxious stimulation or to perform important tasks. Previous studies have shown that performing a task can lead to a modest attenuation of perceived pain intensity (5% to 12%; e.g., [7, 12]) or to no pain attenuation (e.g., [28, 31–32]). By comparison we showed that performing a relatively simple visual task while concurrently monitoring for a pain stimulus attenuated the perceived pain intensity by up to 28% in healthy adults across different stimuli and visual tasks. This attenuation is not too far off from the accepted clinically relevant attenuation level of 30% [66–67]. Moreover, our threat manipulation moderated pain attenuation by almost three times. As Melzack and Casey [68] elegantly stated, pain can be “treated not only by trying to cut down the sensory input by anaesthetic block, surgical intervention and the like, but also by influencing the motivational-affective and cognitive factors as well” (p. 435). As demonstrated here, our novel incremental dual-task paradigm provides a simple yet robust means to further investigate the wide range of “motivational-affective and cognitive factors” which can moderate task engagement for the purpose of attenuating perceived pain intensity. Using this paradigm, future work can systematically investigate what task-related and task-unrelated factors give the greatest affective-motivational value and how much inter-individual variation exists while systematically controlling for attentional processes. Such work will have important implications for developing effective behavioural strategies for managing acute and chronic pain [69–70].

## Supporting information

S1 Text Image selection procedure for Experiment 2. Description of the procedure to select images for Experiment 2.
(DOCX)

S1 File Excel file of data for Experiments 1 and 2. Force and behavioural data for each individual participant in Experiments 1 and 2.
(XLSX)

S2 File Excel file of questionnaire data for image selection in Experiment 2. Questionnaire excel data from Qualtrics for selecting the images in Experiment 2.
(XLSX)
