# Supplementary material for: An incremental dual-task paradigm to investigate pain attenuation by task difficulty, affective content and threat value
Source: PLoS One. 2018 Nov 9;13(11):e0207023. doi: 10.1371/journal.pone.0207023 (PMC6226192; doi:10.1371/journal.pone.0207023)
Supplement: S1 Text — Description of the procedure to select images for Experiment 2. (DOCX) [file pone.0207023.s001.docx]

**Procedure to select images for Experiment 2**

We designed an online questionnaire using Qualtrics to select images with different affective content to be used in Experiment 2. Sixty-three participants (out of 217) completed the questionnaire (51 females; age: 18 – 57 years, *M* = 24.9 years, *SD* = 11.8 years). Their task was to rate 160 images on up to 5 different scales per image (arousal, valance and the three catastrophizing scales; [1]). One hundred twenty images were selected from the International Affective Picture System (IAPS; [2]) and 40 images were downloaded from the internet. We initially selected 40 IAPS images to be “positive” (e.g., a smiling baby), 40 to be “negative” (e.g., a person being threatened by a gun) and 40 to be “neutral” (e.g., a car on the street) based on the content of the images. The remaining 40 images depicted people in different levels of discomfort; these formed a “discomfort” image set. For each participant, the 160 images were presented sequentially in a random order. The participants rated each image on an arousal scale (1 = calm, 9 = excited) and a valence scale (1 = unhappy, 9 = happy; 53). For the discomfort images, participants additionally rated each image on the three catastrophizing scales (1): magnification (how much does the image make you feel like “I wonder whether something serious might happen”? 1 = not much, 9 = very much), rumination (how much does the image make you feel like “I keep thinking about how much it hurts”? 1 = not much, 9 = very much) and helplessness (how much does the image make you feel like “I feel I can’t go on”? 1 = not much, 9 = very much). For the positive image set, we selected 12 images which had an average arousal rating > 4.5 and an average valence rating > 6.5. For the negative image set, we selected 12 images which had an average arousal rating > 3.5 and an average valence rating < 2.0. For the neutral image set, we selected 12 images that had an average arousal rating between 3.0 and 5.0, and an average valence rating between 4.5 and 6.0. Finally, for the discomfort image set, we selected 12 images with an average rating of 4.5 across the 3 catastrophizing scales. These values were arbitrarily set to allow us to select the same number of stimuli in each image set. Table S1 provides the mean valence and arousal ratings averaged across the 12 images in each image set (with the additional catastrophizing scales for the discomfort image set). For the final set of 36 IAPS images used, there was a positive correlation between our average ratings and those reported by Lang et al. ([2]; arousal: *r* = .47; valence: *r* = .99; *p*s < .05).

**Table S1. Results of the questionnaire for image selection.**

|  | Arousal | Valence | Magnification | Rumination | Helplessness |
| --- | --- | --- | --- | --- | --- |
| Positive | 5.3 (.2) | 6.8 (.1) | - | - | - |
| Negative | 4.3 (.1) | 1.8 (.01) | - | - | - |
| Neutral | 3.8 (.2) | 5.2 (.1) | - | - | - |
| Discomfort | 3.0 (.1) | 2.6 (.1) | 4.2 (.1) | 4.5 (.1) | 3.6 (.1) |

The mean (standard deviation) of the arousal and valence scores averaged across the 12 images in each image set used in Experiment 2.

**Supplementary references**

[1] Sullivan MJL, Bishop SP, Pivik J. The Pain Catastrophizing Scale: Development and validation. Psychol Assess. 1995; 7: 524-532.

[2] Lang PJ, Bradley MM, Cuthbert BN. International affective picture system (IAPS): Affective ratings of pictures and instruction manual. Technical Report A-6. 2005; University of Florida: Gainesville, 1-54.
